# Supplementary material for: Modeling glioblastoma heterogeneity as a dynamic network of cell states
Source: Mol Syst Biol. 2021 Sep 16;17(9):e10105. doi: 10.15252/msb.202010105 (PMC8444284; doi:10.15252/msb.202010105)
Supplement: Supplementary file 6 — Source Data for Figure 5 [file MSB-17-e10105-s004.zip › Figure5A_sourcedata/GSEA_3071/nosigdb_stateVI.GseaPreranked.1622795169056/NB_KI_CLUSTER11.html]

Details for gene set NB\_KI\_CLUSTER11[GSEA]

|  || Dataset | state63071 |
| Phenotype | NoPhenotypeAvailable |
| Upregulated in class | na\_pos |
| GeneSet | NB\_KI\_CLUSTER11 |
| Enrichment Score (ES) | 0.33997983 |
| Normalized Enrichment Score (NES) | 1.7804415 |
| Nominal p-value | 0.016736401 |
| FDR q-value | 0.018803898 |
| FWER p-Value | 0.176 |
Table: GSEA Results Summary

  

Fig 1: Enrichment plot: NB\_KI\_CLUSTER11      
 Profile of the Running ES Score & Positions of GeneSet Members on the Rank Ordered List

  

| PROBE | GENE SYMBOL | GENE\_TITLE | RANK IN GENE LIST | RANK METRIC SCORE | RUNNING ES | CORE ENRICHMENT || 1 | THBS1 |  |  | 12 | 1.097 | 0.0625 | Yes |
| 2 | PLAUR |  |  | 34 | 0.936 | 0.1128 | Yes |
| 3 | IL32 |  |  | 46 | 0.880 | 0.1625 | Yes |
| 4 | GLIPR1 |  |  | 81 | 0.781 | 0.1999 | Yes |
| 5 | PPP1R15A |  |  | 98 | 0.738 | 0.2397 | Yes |
| 6 | MYL12A |  |  | 173 | 0.627 | 0.2568 | Yes |
| 7 | CLIC1 |  |  | 258 | 0.561 | 0.2671 | Yes |
| 8 | SH3BGRL3 |  |  | 265 | 0.556 | 0.2988 | Yes |
| 9 | SP100 |  |  | 294 | 0.544 | 0.3236 | Yes |
| 10 | PFN1 |  |  | 347 | 0.513 | 0.3400 | Yes |
| 11 | FOSL2 |  |  | 717 | 0.393 | 0.2613 | No |
| 12 | IRF1 |  |  | 771 | 0.382 | 0.2695 | No |
| 13 | CIB1 |  |  | 807 | 0.375 | 0.2822 | No |
| 14 | KLF2 |  |  | 814 | 0.374 | 0.3030 | No |
| 15 | ARPC1B |  |  | 847 | 0.369 | 0.3162 | No |
| 16 | SDC4 |  |  | 1013 | 0.343 | 0.2911 | No |
| 17 | CCND2 |  |  | 1064 | 0.335 | 0.2973 | No |
| 18 | ANXA11 |  |  | 1094 | 0.331 | 0.3091 | No |
| 19 | S100A6 |  |  | 1193 | 0.317 | 0.3010 | No |
| 20 | EMP3 |  |  | 1278 | 0.306 | 0.2960 | No |
| 21 | FLNA |  |  | 1737 | 0.257 | 0.1845 | No |
| 22 | CTSC |  |  | 1767 | 0.254 | 0.1917 | No |
| 23 | SAT1 |  |  | 1952 | -0.271 | 0.1569 | No |
| 24 | ARID5B |  |  | 2156 | -0.306 | 0.1190 | No |
| 25 | B2M |  |  | 2460 | -0.369 | 0.0571 | No |
| 26 | EEF1D |  |  | 2480 | -0.373 | 0.0741 | No |
| 27 | NFE2L2 |  |  | 2707 | -0.427 | 0.0371 | No |
| 28 | RARRES3 |  |  | 2942 | -0.510 | 0.0028 | No |
| 29 | PNRC1 |  |  | 3055 | -0.566 | 0.0057 | No |
| 30 | GYPC |  |  | 3169 | -0.638 | 0.0126 | No |
| 31 | TGIF1 |  |  | 3380 | -0.860 | 0.0059 | No |
| 32 | LITAF |  |  | 3491 | -1.089 | 0.0408 | No |
Table: GSEA details [plain text format]

  

Fig 2: NB\_KI\_CLUSTER11: Random ES distribution      
 Gene set null distribution of ES for **NB\_KI\_CLUSTER11**
